# Adverse effects from antidepressant treatment: randomised controlled trial of 601 depressed individuals

**DOI:** 10.1007/s00213-014-3467-8

**Published:** 2014-02-13

**Authors:** Andrew A. Crawford, Sarah Lewis, David Nutt, Tim J. Peters, Philip Cowen, Michael C. O’Donovan, Nicola Wiles, Glyn Lewis

**Affiliations:** 1 School of Social and Community Medicine, University of Bristol, Oakfield House, Oakfield Grove, Bristol, BS8 2BN UK; 2 Department of Neuropsychopharmacology, Imperial College, London, UK; 3 School of Clinical Sciences, University of Bristol, Bristol, UK; 4 Department of Psychiatry, Warneford Hospital, University of Oxford, Oxford, UK; 5 MRC Centre for Neuropsychiatric Genetics and Genomics, School of Medicine, Cardiff University, Cardiff, UK; 6 Mental Health Sciences Unit, University College London, London, UK

**Keywords:** Adverse effects, Antidepressants, Citalopram, Depression, Noradrenaline reuptake inhibitor, NARI, Selective serotonin reuptake inhibitor, SSRI, Reboxetine

## Abstract

**Rationale:**

Premature discontinuation of antidepressant drugs is a frequent clinical problem. Adverse effects are common, occur early on in treatment and are reported to be one of the main reasons for discontinuation of antidepressant treatment.

**Objectives:**

To investigate the association between adverse effects occurring in the first 2 weeks of antidepressant treatment and discontinuation by 6 weeks as the outcome. To investigate the time profile of adverse effects induced by the selective serotonin reuptake inhibitor citalopram and the noradrenaline reuptake inhibitor reboxetine over 12 weeks of treatment.

**Methods:**

Six hundred and one depressed individuals were randomly allocated to either citalopram (20 mg daily) or reboxetine (4 mg twice daily). A modified version of the Toronto Side Effects Scale was used to measure 14 physical symptoms at baseline (medication free) and at 2, 6 and 12 weeks after randomisation.

**Results:**

Individuals randomised to reboxetine reported a greater number of adverse effects and were more likely to stop treatment than individuals receiving citalopram. Dizziness (OR 1.83; 95% CI 1.09, 3.09; *p* = 0.02) and the total number of adverse effects (OR 1.12; 95% CI 1.00, 1.25; *p* = 0.06) reported at 2 weeks were associated with discontinuation from overall antidepressant treatment by 6 weeks. Reports of adverse effects tended to reduce throughout the 12 weeks for both antidepressants.

**Conclusions:**

The majority of adverse effects were not individually associated with discontinuation from antidepressant treatment. Reports of physical symptoms tended to reduce over time. The physical symptoms that did not reduce over time may represent symptoms of depression rather than antidepressant-induced adverse effects.

**Electronic supplementary material:**

The online version of this article (doi:10.1007/s00213-014-3467-8) contains supplementary material, which is available to authorized users.

## Introduction

Depression is a chronic disorder affecting a relatively high proportion of individuals each year (Wittchen et al. 2011). Long-term treatment is necessary to alleviate the symptoms of depression. Antidepressants are effective at treating depression, but discontinuation rates are high (Dunn et al. 1999). Adverse effects occur frequently with antidepressant medication (Cramer and Rosenheck 1998), especially early in treatment (Bull et al. 2002), and are reported as a common reason for early discontinuation from antidepressant therapy (Demyttenaere et al. 2001).

Selective serotonin reuptake inhibitors (SSRIs) are the most commonly prescribed antidepressants in the UK, and citalopram accounted for over half of all SSRI prescriptions in 2011 (13.5 million prescriptions) (NHS 2011). Despite their superior adverse effect profile compared with the older generation of tricyclic antidepressants (TCAs), SSRIs frequently cause gastrointestinal adverse effects, insomnia, headache, anxiety and sexual dysfunction (Goldstein and Goodnick 1998; Ferguson 2001a). TCAs are less well tolerated than either SSRIs or selective noradrenaline reuptake inhibitors (NARIs) potentially due to their broader pharmacologic mechanism of action (Anderson 2000). NARIs act specifically via the noradrenaline transporter, leading to an increase in synaptic noradrenaline. Reboxetine was the first NARI to be licenced in the UK and accounted for just 0.1 % of the total annual prescriptions in 2011 (NHS 2011). It has been reported that reboxetine is less well tolerated than citalopram and can induce adverse effects such as dry mouth, constipation, increased sweating and hot flushes (Hajos et al. 2004; Cipriani et al. 2009; Eyding et al. 2010).

Previous studies have reported rates of discontinuation of antidepressant medication ranging from 33 to 53 % with adverse effects cited as the reason for 23–36 % of discontinuations (Demyttenaere et al. 2001; Hu et al. 2004). Others have reported more discontinuations among individuals randomised to reboxetine than to citalopram (49 % vs. 31 % for citalopram) (Langworth et al. 2006), and adverse effects were cited more frequently as the reason for discontinuation from reboxetine (40 % vs. 17 % for citalopram) (Langworth et al. 2006).

There are fewer data on whether specific adverse effects are associated with discontinuation from antidepressant treatment. In the genome-based therapeutic drugs for depression (GENDEP) study, 811 adults with moderate to severe unipolar depression were either randomised to receive escitalopram (SSRI) or nortriptyline, or, if contraindications for one of the drugs were present, allocated non-randomly to the other antidepressant. Nortriptyline is a TCA that predominantly inhibits noradrenaline reuptake. The authors reported evidence that urinary problems, dryness of mouth, blurred vision and orthostatic dizziness, as well as the total number of adverse effects reported, were associated with discontinuation from overall antidepressant treatment (Uher et al. 2009a).

Several studies have reported that adverse effects progressively decrease over time as the person continues to take the medication (Greist et al. 2004; Uher et al. 2009a). The evidence-based guidelines for treating depressive disorders with antidepressants, compiled by the British Association for Psychopharmacology (BAP), report that adverse effects tend to improve over time; moreover, some of these effects (such as nausea on SSRIs) are usually short-lived while others (such as anticholinergic adverse effects on TCAs) appear to be more resilient (Anderson et al. 2008). Since physical symptoms are common in depression, the identification of adverse effects requires judgement on the part of the patient (Haug et al. 2004).

We report on the adverse effect profile following treatment with citalopram or reboxetine over a 12-week period. We investigated how reports of specific adverse effects changed over the 12 weeks of treatment. In addition, we examined whether adverse effects occurring early during the course of antidepressant treatment (in the first 2 weeks) are associated with discontinuation of medication.

## Methods

### The GENPOD trial

The trial methods have been reported in detail elsewhere (Thomas et al. 2008). In brief, the study was a multicentre, randomised controlled trial (RCT) conducted in three UK centres (Bristol, Birmingham and Newcastle) in which patients with depression, recruited in primary care, were randomly allocated to receive either citalopram (20 mg daily) or reboxetine (4 mg twice daily). The genetic and clinical predictors of treatment response in depression (GENPOD) trial were designed to test two primary hypotheses regarding genetic and clinical predictors of response to antidepressant medication (Lewis et al. 2011; Wiles et al. 2012). Secondary analysis of these trial data can nevertheless provide information on the adverse effect profile of these two antidepressants.

Eligible participants were those aged 18–74 years who had already agreed with their general practitioner (GP) that an antidepressant should be prescribed. Patients who had taken antidepressant medication within the 2 weeks prior to the baseline assessment and those who could not complete self-administered questionnaires were excluded. GPs also excluded those with medical contraindications to antidepressant medication, those who had a history of psychosis, bipolar affective disorder, major substance or alcohol misuse and others whose participation was deemed inappropriate. At the baseline assessment, only patients with depression that fulfilled the ICD-10 criteria for depression (category F32), assessed using computerised Clinical Interview Schedule—revised version (CIS-R) (Lewis et al. 1992; WHO 1992; Lewis 1994), and a Beck Depression Inventory (BDI) (Beck et al. 1961) score above 14, were eligible to participate in the study.

#### Randomisation procedure

Following the baseline assessment, eligible patients were asked to provide written informed consent for trial participation. Randomisation was conducted using a computer-generated code, administered centrally and communicated by telephone and thereby concealed in advance from the researcher. Allocation was stratified by severity of depressive symptoms (CIS-R total score ≥28 or <28) and by centre, using variable block sizes to maximise concealment. The researcher gave the randomised medication to the participant. Those randomly allocated to reboxetine were advised to begin with a dose of 2 mg twice daily and increase it to 4 mg twice daily after about 4 days. Individuals randomised to citalopram received 20 mg daily. All patients were advised that they could approach their GP if they wished to increase the dose of their allocated treatment. Patients, GPs and researchers were not blinded to treatment allocation.

#### Measures

Self-reported outcome data on physical symptoms were collected at baseline and at 2, 6 and 12 weeks after randomisation. Data were collected over the telephone at 2 weeks and by means of a self-administered questionnaire at baseline, 6 and 12 weeks. A modified version of the Toronto Side Effects Scale (Vanderkooy et al. 2002) was used to measure 14 physical symptoms (Supplementary Figure 1). Individuals reported the number of days that they had experienced each physical symptom in the last week (0, 1–3, 4–7 days). A measure of baseline physical symptoms was required in order to identify the occurrence of adverse effects (physical symptoms induced by antidepressant treatment), rather than residual symptoms of depression. Physical symptoms reported at 2, 6 and 12 weeks will be referred to as adverse effects from this point on. Participants were only asked about the presence and frequency of physical symptoms and were not asked whether the symptom was caused by the antidepressant. Discontinuation of antidepressant medication was assessed at 6 and 12 weeks by self report. Adherence to medication was also assessed at 6 weeks by a pill count of returned medication.

#### Statistical analysis

Data were analysed using Stata version 12.1 (StataCorp 2011). In preliminary analyses, relatively few individuals reported suffering from the adverse effects for four or more days in the previous week; therefore, data on individual adverse reactions were collapsed into dichotomous variables (present/absent) for further analyses. A measure of general adverse effect burden was derived from the number of adverse effects reported at each time point and treated as a continuous variable (not including the male-specific questions). All analyses were adjusted for physical symptoms reported at baseline (medication free) so that only adverse effects induced by citalopram or reboxetine were investigated.

To investigate whether the two antidepressants had distinct adverse effect profiles, we compared reports of adverse effects between individuals allocated to citalopram and reboxetine at 2, 6 and 12 weeks after randomisation using random effects logistic or linear regression models to account for repeated measurements for each individual. Summary odds ratios (OR), 95 % confidence intervals (95 % CI) and *p* values were reported. We formally evaluated the appropriateness of this summary measure by including an interaction term between time and randomised treatment.

Random effects logistic (for individual adverse effects) and linear (for the number of adverse effects reported) regression models using data from 0 to 2 weeks in a repeated measures model were used to examine whether reports of physical symptoms increased from baseline (medication free) to 2 weeks after receiving treatment. Random effects logistic and linear regression models using data from 2, 6 and 12 weeks in a repeated measures model were used to examine whether the reporting of adverse effects changed over the 12 weeks. We investigated the effect of time in these models, as described earlier.

Logistic regression models were used to investigate whether adverse effects at 2 weeks are associated with discontinuation from antidepressant treatment by 6 weeks. We investigated whether the impact of the adverse effect varied by treatment group by including an interaction term between adverse effect and randomised treatment.

As has been reported previously (Wiles et al. 2012), individuals lost to follow-up at 6 weeks were younger and reported more life events at baseline. The impact of missing data on our findings was investigated by adjusting for these factors in the various regression models. This method should address any bias under a missing at random assumption (Carpenter and Kenward 2013). Sensitivity analyses were conducted in the subset of those individuals who adhered to treatment. All models were adjusted for the trial ‘design’ (stratification) variables (severity of baseline depression (CIS-R score <28 and ≥ 28) and recruitment centre) and, where appropriate, treatment allocation.

## Results

### Trial participation and follow-up

A comparison of the randomised groups at baseline and the CONSORT flow chart for the GENPOD trial has previously been published (Lewis et al. 2011). In total, 601 participants (68 % female, *n* = 408) were randomised to receive either citalopram (*n* = 298) or reboxetine (*n* = 303). The average participant was aged 38.8 years (sd 12.4) with a baseline BDI score of 33.7 (sd 9.7) and CIS-R score of 30.8 (sd 8.0).

Fifty six participants randomised to citalopram had their daily dose increased from 20 mg to 30 mg (*n* = 11), 40 mg (*n* = 33) and 60 mg (*n* = 11) (one further participant had an increase in the prescribed dose of an unknown amount). Thirteen of those allocated to reboxetine had their daily dose increased to 10 mg (*n* = 3), 12 mg (*n* = 9) and 16 mg (*n* = 1). Ninety-six percent (*n* = 576) completed the 2-week follow-up (citalopram *n* = 284, reboxetine *n* = 292), 91 % (*n* = 546) completed the 6-week follow-up (citalopram *n* = 274, reboxetine *n* = 272) and 81 % (*n* = 486) completed the 12-week follow-up (citalopram *n* = 253, reboxetine *n* = 233).

### Data completeness

For the majority of adverse effects, there were complete data in over 99 % of the individuals followed up. There was a greater amount of missing data for the questions relating to male-specific adverse effects. For the question referring to difficulty with ejaculation, 75 % (*n* = 145) of males had complete data at baseline, 81 % (*n* = 150) at 2 weeks, 86 % (*n* = 149) at 6 weeks and 84 % (*n* = 123) at 12 weeks. Similarly, for impotence, 80 % (*n* = 154) had complete data at baseline, 86 % (*n* = 160) at 2 weeks, 86 % (*n* = 149) at 6 weeks and 85 % (*n* = 124) at 12 weeks.

### Discontinuation from antidepressant treatment

A greater proportion of individuals discontinued treatment from reboxetine than citalopram (53 % vs. 31 %, *p* < 0.001) over the 12 weeks. Thirteen percent (*n* = 77) of all individuals discontinued antidepressant treatment by the 2-week follow-up (citalopram *n* = 27, reboxetine *n* = 50), 26 % (*n* = 159) discontinued by the 6-week follow-up (citalopram *n* = 51, reboxetine *n* = 108) and 42 % (*n* = 255) discontinued by the 12-week follow-up (citalopram *n* = 93, reboxetine *n* = 162). The most common reason for discontinuation was due to adverse effects reported by 61 %, 57 % and 43 % of individuals who discontinued at 2, 6 and 12 weeks, respectively.

### Frequencies of adverse effects and drug comparisons

The majority of adverse effects reported at 2 weeks were actually less common than reports of the physical symptoms at baseline (when individuals were not taking an antidepressant, ‘medication free’) (Supplementary Table 4). For example, an individual had 32 % lower odds of reporting tremor after 1 week of treatment compared to baseline (OR 0.68, 95% CI 0.58, 0.81; *p* < 0.001). However, individuals randomised to reboxetine reported dry mouth, constipation and flushing, as well as impotence and problems with ejaculation for males, more commonly at 2 weeks than at baseline. There was also some evidence to suggest that males randomised to citalopram reported problems with ejaculation more commonly at 2 weeks than at baseline. When analysis was restricted to only include individuals who adhered to treatment or included factors associated with missing data in the regression model, the results were consistent with the main analysis (data not shown).

**Fig. 1:**
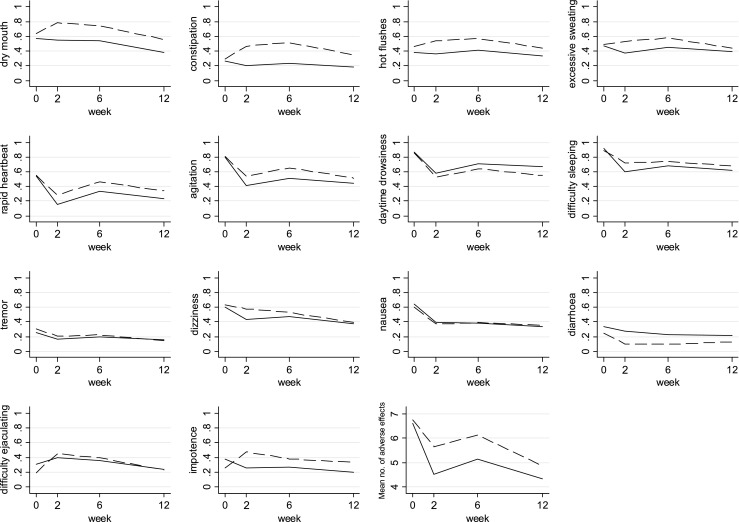
Plots showing proportion of individuals reporting each adverse effect and the mean number of adverse effects over the 12-week trial period. Plots show the proportion of individuals reporting each adverse effect at baseline and at 2, 6 and 12 weeks after randomisation to citalopram or reboxetine. The *solid line* represents individuals receiving citalopram. The *dashed line* represents individuals receiving reboxetine. The plots for difficulty ejaculating and impotence show the proportion of men who reported these adverse effects. The plot ‘number of adverse effects’ shows the mean number of adverse effects

The proportion of individuals reporting each physical symptom at baseline and each adverse effect at 2, 6 and 12 weeks after randomisation can be seen graphically in Fig. 1, and the exact figures for those allocated to citalopram in Supplementary Table 1 and to reboxetine in Supplementary Table 2. The most frequently reported adverse effects amongst those allocated to citalopram were daytime drowsiness (71 % (*n* = 193) of individuals after 6 weeks) and difficulty sleeping (68 % (*n* = 186) of individuals after 6 weeks). The most frequently reported adverse effects amongst those allocated to reboxetine were dry mouth, which was reported by 78 % (*n* = 228) of individuals after 2 weeks, and difficulty sleeping, which was reported by 74 % (*n* = 200) of participants after 6 weeks.

On average, the number of adverse effects reported by individuals randomised to reboxetine was 5.6, 6.1 and 4.9 at 2, 6 and 12 weeks, respectively. Fewer adverse effects were reported in the citalopram group (4.5, 5.2 and 4.3 adverse effects reported at the same time points) (Supplementary Tables 1 and 2).

To investigate whether the two antidepressants have distinct adverse effect profiles, we compared reports of adverse effects between individuals allocated to citalopram and reboxetine at 2, 6 and 12 weeks after randomisation in a repeated measures analysis (Table 1). Nine of the 14 adverse effects were more commonly reported during treatment with reboxetine. The odds of reporting constipation, over the 12 weeks of treatment, were six times higher for individuals receiving reboxetine than citalopram. Only diarrhoea and daytime drowsiness were less commonly reported with reboxetine treatment, with no evidence of a difference in reports of tremor, nausea or difficulty ejaculating.

**Table 1:** Summary odds ratios or linear regression coefficient of reporting adverse effects amongst those allocated to receive reboxetine compared with those allocated to receive citalopram from a repeated measures analysis at 2, 6 and 12 weeks

Adverse Effect	*N*	OR	95 % CI	*p* value	*p* value for interaction between treatment and time
Tremor	1,608	0.99	0.63, 1.58	0.98	0.12
Agitation	1,607	1.76	1.33, 2.33	<0.001	0.28
Dry mouth	1,603	3.33	2.25, 4.91	<0.001	0.09
Excessive sweating	1,607	1.89	1.33, 2.69	<0.001	0.02
Constipation	1,606	6.09	3.76, 9.87	<0.001	0.08
Diarrhoea	1,605	0.28	0.18, 0.45	<0.001	0.05
Nausea	1,608	1.04	0.76, 1.40	0.82	0.47
Dizziness	1,607	1.50	1.06, 2.11	0.02	0.02
Daytime drowsiness	1,608	0.59	0.41, 0.85	0.005	0.22
Difficulty sleeping	1,605	1.90	1.26, 2.86	0.002	0.19
Hot Flushes	1,606	2.59	1.64, 4.07	<0.001	0.08
Rapid heartbeat	1,607	2.51	1.65, 3.83	<0.001	0.33
Impotence	373	5.21	1.96, 13.85	0.001	0.60
Difficulty ejaculating	344	1.48	0.48, 4.57	0.49	0.80
	*N*	Coefficient	95% CI	*p* value	*p* value for interaction between treatment and time
Number of adverse effects	1,597	0.79	0.48, 1.10	<0.001	0.007

The comparison between the two antidepressants includes all individuals randomly allocated to receive either reboxetine or citalopram. Models are adjusted for baseline adverse effect, severity of depression and centre. An OR greater than 1 indicates, that over the 12 weeks, the adverse effect was more commonly reported by individuals randomised to reboxetine than citalopram. A positive coefficient indicates a greater number of adverse effects reported by individuals randomised to reboxetine than citalopram over the 12 weeks. The interaction reports the *p* value for the interaction between time (as a continuous variable) and allocated treatment group

For two of the adverse effects, and for the total number of adverse effects reported, there was evidence that the effect of the treatment varied with time (*p* value for interaction between treatment allocation and time is <0.05) (Table 2). There was a weaker evidence of an interaction between treatment allocation and time for a further four adverse effects (*p* value < 0.1). In all cases, the effect of the drug on adverse effects was less pronounced at 12 weeks than at 2 weeks. For example, on average, individuals allocated to reboxetine reported one more adverse effect at 2 weeks (OR 1.08, 95% CI 0.72, 1.45; *p* < 0.001) but only 0.4 more at 12 weeks (OR 0.41, 95% CI −0.06, 0.88; *p* = 0.08) compared to individuals allocated to citalopram. The time-specific ORs or coefficients at 2, 6 and 12 weeks for these seven outcomes are reported in Supplementary Table 3.

**Table 2:** Summary odds ratios or linear regression coefficients reporting the effect of time on adverse effect reporting in a combined analysis, and stratified by allocated treatment group from a repeated measures analysis at 2, 6 and 12 weeks

Adverse effect	Citalopram			Reboxetine			Combined		
	OR	95 % CI	*p* value	OR	95 % CI	*p* value	*p* value for interaction between time and treatment, *p*	OR	95 % CI
Tremor	0.99	0.94, 1.05	0.82	0.93	0.88, 0.99	0.02	0.12	0.96	0.92, 1.00
Agitation	1.01	0.97, 1.05	0.56	0.98	0.94, 1.02	0.35	0.28	1.00	0.97, 1.02
Dry mouth	0.89	0.85, 0.94	<0.001	0.86	0.82, 0.90	<0.001	0.09	0.88	0.85, 0.91
Excessive sweating	1.01	0.97, 1.05	0.68	0.94	0.90, 0.98	0.003	0.02	0.97	0.94, 1.00
Constipation	0.97	0.92, 1.02	0.28	0.91	0.87, 0.95	<0.001	0.08	0.94	0.90, 0.97
Diarrhoea	0.95	0.91, 1.00	0.06	1.04	0.97, 1.11	0.29	0.05	0.98	0.94, 1.02
Nausea	0.97	0.93, 1.01	0.10	0.99	0.95, 1.03	0.56	0.47	0.98	0.95, 1.01
Dizziness	0.96	0.92, 1.00	0.05	0.90	0.86, 0.94	<0.001	0.02	0.93	0.90, 0.95
Daytime drowsiness	1.05	1.01, 1.10	0.02	1.01	0.97, 1.05	0.61	0.22	1.03	1.00, 1.06
Difficulty sleeping	1.01	0.96, 1.06	0.67	0.97	0.93, 1.02	0.19	0.19	0.99	0.96, 1.02
Hot Flushes	0.97	0.93, 1.02	0.29	0.92	0.88, 0.97	0.001	0.08	0.95	0.91, 0.98
Rapid heartbeat	1.06	1.01, 1.11	0.03	1.02	0.98, 1.07	0.33	0.33	1.04	1.00, 1.07
Impotence	0.91	0.81, 1.03	0.15	0.89	0.80, 0.99	0.03	0.60	0.90	0.83, 0.98
Difficulty ejaculating	0.79	0.68, 0.93	0.003	0.85	0.76, 0.96	0.007	0.80	0.83	0.76, 0.91
	Coefficient	95 % CI	*p* value	Coefficient	95 % CI	*p* value	*p* value for interaction between time and treatment, *p*	Coefficient	95 % CI
Number of adverse effects	−0.03	−0.06, 0.00	0.1	−0.09	−0.13, −0.05	<0.001	0.007	−0.06	−0.08, −0.03

The summary odd ratios indicate the effect of an increase in time of 1 week on reports of each adverse effect. Models are adjusted for severity of depression, centre and baseline adverse effect and in the combined analysis, allocated treatment. An OR greater than 1 (or positive coefficient) indicates that the adverse effect increases with time. The interaction reports the *p* value for the interaction between time (as a continuous variable) and allocated treatment group

### Time course of adverse effects

Collection of data at baseline and 2, 6 and 12 weeks after randomisation to medication allowed us to determine the time course of adverse effects over the treatment period. Reports of seven adverse effects (tremor, dry mouth, constipation, dizziness, hot flushes, impotence, difficulty ejaculating), as well as the number of adverse effects reported, reduced over the 12 weeks for both antidepressants (Table 2). There was a weaker evidence that nausea was also reported less frequently with time. Reports of agitation, difficulty sleeping, drowsiness and rapid heartbeat initially increased between 2 and 6 weeks and then decreased from 6 to 12 weeks (Fig. 1). There was a difference in how reports of sweating (*p* value for interaction between time and treatment, *p* = 0.02), dizziness (*p* = 0.02) and diarrhoea (*p* = 0.05) changed over the 12 weeks for the two antidepressants. Reports of sweating only reduced over time for individuals receiving reboxetine, while reports of diarrhoea only reduced for individuals receiving citalopram (Table 2). For example, the odds of reporting diarrhoea in the citalopram group decreased by 5 % for every week that passed (OR 0.95, 95% CI 0.91, 1.00), while in the reboxetine group, they did not (OR 1.04, 95% CI 0.97, 1.11). When analysis was restricted to individuals who adhered to treatment, the results were not substantially different (Supplementary Table 5).

### Are adverse effects reported at 2 weeks associated with discontinuation by 6 weeks?

There was evidence of an association between antidepressant-induced dizziness reported at 2 weeks and discontinuation of antidepressant treatment by 6 weeks (OR 1.83, 95% CI 1.09, 3.09; *p* = 0.02). There was also evidence that every additional adverse effect reported at 2 weeks was associated with a 12 % higher odds of discontinuation by 6 weeks (OR 1.12, 95% CI 1.00, 1.25; *p* = 0.06). There was no evidence of an association between any other adverse effect and discontinuation (Table 3). Additionally, there was no evidence that the impact of the adverse effect on discontinuation was different between the two treatment groups (interaction term between adverse effect and randomised treatment, *p* > 0.1). However, the results stratified by treatment are available in Supplementary Table 6. When the analysis was restricted to only include individuals who adhered to treatment at 2 weeks or included factors associated with missing data, the results were consistent with the main analysis (data not shown).

**Table 3:** Odds ratios of discontinuing antidepressant treatment between 2 and 6 weeks by adverse effect at 2 weeks

Adverse effect at 2 weeks	*N*	OR	95 % CI	*p* value	*p* value for interaction between adverse effect and treatment, *p*
Tremor	512	1.37	0.73, 2.55	0.32	0.52
Agitation	512	1.47	0.90, 2.41	0.13	0.26
Dry mouth	511	1.00	0.55, 1.79	0.99	0.35
Excessive sweating	512	1.39	0.85, 2.29	0.19	0.71
Constipation	511	1.42	0.85, 2.38	0.19	0.17
Diarrhoea	512	1.25	0.63, 2.45	0.52	0.16
Nausea	512	1.45	0.89, 2.36	0.14	0.12
Dizziness	512	1.83	1.09, 3.09	0.02	0.85
Daytime drowsiness	512	0.69	0.43, 1.12	0.14	0.14
Difficulty sleeping	512	0.86	0.50, 1.48	0.60	0.38
Hot flushes	512	1.51	0.90, 2.51	0.12	0.63
Rapid heartbeat	512	1.12	0.64, 1.98	0.69	0.58
Impotence	121	0.94	0.33, 2.67	0.91	0.71
Difficulty ejaculating	109	1.34	0.48, 3.72	0.58	0.92
Number of adverse effects	510	1.12	1.00, 1.25	0.06	0.63

Odds ratios (OR) are estimates from logistic regression models with discontinuation from treatment as the outcome. Models are adjusted for baseline adverse effect, allocated treatment, severity of depression and centre. An OR greater than 1 indicates that the adverse effect at 2 weeks is associated with a higher odds of discontinuation from antidepressant treatment between 2 and 6 weeks. The interaction reports the *p* value for the interaction between adverse effect and allocated treatment group

## Discussion

### Main findings

To our knowledge, GENPOD is the largest RCT of depressed individuals receiving the pharmacologically different antidepressants, citalopram and reboxetine. The adverse effects reported by individuals in GENPOD are largely in agreement with the adverse effect profiles previously reported (Ferguson 2001a; Hajos et al. 2004; Langworth et al. 2006). Additionally, participants allocated to receive reboxetine reported a greater number of adverse effects and were more likely to discontinue antidepressant treatment. The impact of adverse effects on discontinuation was the same for both antidepressants, and only reports of antidepressant-induced dizziness were associated with increased odds of discontinuation by 6 weeks. Reports of the majority of physical symptoms were more common at baseline (medication free) than reports of adverse effects when on medication. In general, reports of adverse effects tended to reduce throughout the 12 weeks.

### Strengths and limitations

The main strengths of the study are the large sample size and the high follow-up rates (91 % at 6 weeks). Nevertheless, even a small amount of missing data has the potential to introduce bias. However, adjustment for factors associated with missing data produced results consistent with our main analysis. Additionally, we were able to assess the effect of adherence by restricting analyses to include only the individuals who were taking their medication. Assessing adherence by self report is not the most accurate method; however, it is likely that any errors would be the same in both treatment groups resulting in a loss of precision, but not a source of bias.

Different methodologies were used to record physical symptom data over the course of the trial potentially confounding our results. Data were collected over the telephone at 2 weeks and by means of a self-administered questionnaire at baseline, 6 and 12 weeks. However, the comparison of face-to-face and telephone assessments for mental health measures has suggested that the mode of administration does not introduce any bias (Evans et al. 2004).

We must interpret our results with caution as we were investigating a total of 14 adverse effects and so are at greater risk of obtaining type I errors than when investigating a single exposure. In addition, despite the relatively large sample size, it is possible that we did not have sufficient power to detect interactions, which may have resulted in type II errors; however, we have used the confidence intervals obtained to guide our interpretation.

A placebo arm was not included in the trial which made GENPOD more amenable to individuals actively seeking antidepressant treatment. Therefore, we are limited in only being able to investigate the differences between the two antidepressants, a prototypical SSRI and prototypical noradrenaline reuptake blocking antidepressant or NARI. It is possible that the lack of blinding may have introduced a source of bias as the participants knew which drug they were allocated to. However, this would only occur if the participants had strong preconceptions about the different adverse effect profiles of citalopram and reboxetine, and so, this is unlikely to have influenced the results. The GENPOD trial was also designed to investigate the adverse effect profiles as one of its secondary outcomes (Thomas et al. 2008).

As has been reported previously (Wiles et al. 2012), due to the similar scores on depressive scales and similar response rates to antidepressant treatment seen in other UK depression trials (Ward et al. 2000; Kessler et al. 2009) and the US STAR*D study (Trivedi et al. 2006), the GENPOD sample is a representative of a typical sample of primary care patients with depression. Additionally, both treatment groups were allocated antidepressants prescribed at standard doses, and the GPs retained responsibility for patient care throughout. Therefore, we suggest that our findings are relevant to other European and US populations of people with depression.

### Comparisons with existing literature

#### Physical symptoms at baseline

The majority of physical symptoms were more commonly reported at baseline (medication free) than when actually on medication. This suggests that our instrument used to record adverse effects was also measuring active symptoms of depression. This shows how important it was to include baseline physical symptoms in our analyses to specifically identify reports of new physical symptoms (adverse effects). There were four physical symptoms (dry mouth, constipation, hot flushes and impotence) reported more frequently at 2 weeks than at baseline, and more frequently by those allocated to reboxetine than to citalopram. This suggests that these are specific adverse effects induced by reboxetine, while other differences in the adverse effect profile of the two antidepressants might be due to their differential efficacy in treating specific depressive symptoms. High levels of physical symptoms at baseline have been reported previously, and attempting to distinguish symptoms of depression from antidepressant-induced adverse effects remains a challenge (Moeller 2001; Uher et al. 2009a, b).

#### Adverse effect profile

The adverse effect profiles of citalopram and reboxetine have been well reported and are in general agreement with our findings in GENPOD (Ferguson 2001a; Hajos et al. 2004; Langworth et al. 2006). SSRI-induced adverse effects are thought to occur due to the unwanted stimulation of serotonin receptors. The majority of serotonin receptors are G-protein coupled receptors that activate an intracellular messenger cascade, the exception being the 5-HT3 receptor which is a ligand-gated ion channel. The 5-HT3 receptor was suggested to be a possible mediator of SSRI-induced gastrointestinal adverse effects as 5-HT3 antagonists have been used in the prevention and treatment of nausea, vomiting and diarrhoea (Bailey et al. 1995; Thompson 2013). This would explain the reason why we found evidence that diarrhoea was associated with citalopram treatment, but not our lack of evidence for an association with reports of nausea.

The general consensus is that sexual dysfunction is more commonly reported as an adverse effect induced by SSRI treatment, potentially due to the stimulation of 5-HT2C receptors (Ferguson 2001b; Morehouse et al. 2011). However, we report that impotence was more commonly reported by males allocated to receive reboxetine. Our finding is supported by a study of 450 depressed individuals randomised to three treatment groups, reboxetine, fluoxetine (an SSRI) or placebo, that reported a greater number of problems relating to male arousal, assessed by dichotomous responses, in the reboxetine group (Clayton et al. 2003; Schweitzer et al. 2009). The effects on male arousal may be due to increased levels of noradrenaline stimulating α1-adrenergic receptors (Andersson 2011). These receptors are involved in the contraction of the corpora cavernosa and penile vessels which determine the functional state of the penis.

Adverse effects induced by NARIs are likely due to the stimulation of at least four noradrenergic receptor subtypes in the brain and body. Increased noradrenergic activity at α1-adrenergic receptors may produce symptoms indicative of ‘anticholinergic-type’ adverse effects such as dryness of the mouth and constipation. Reportedly, this is not due to direct blockade of muscarinic cholinergic receptors but instead, due to the indirect reduction of net parasympathetic tone due to increased sympathetic tone, similar to that found in high arousal states such as public speaking (Gruenberg 2009).

#### Discontinuation

Regardless of the antidepressant, we found evidence that the impact of adverse effects on discontinuation was the same. In general, there was a tendency towards adverse effects being associated with higher odds of discontinuation (ie. OR > 1). However, when considering the 95 % CIs, dizziness was the only adverse effect to be associated with higher odds of discontinuation. The GENDEP study also reported an association between dizziness and discontinuation from antidepressant treatment (Uher et al. 2009a).

Dizziness may have a greater impact on daily routine (that is, on driving) than other adverse effects which may explain why we found evidence of an association with discontinuation. The cause of antidepressant-induced dizziness is not known; in the case of reboxetine, it might be postural hypotension from altered baroreceptor function as found with other noradrenaline-acting TCA antidepressant imipramine (Middleton et al. 1988). SSRI-induced dizziness is much less explored but may reflect a direct effect on the inner ear. Informing patients of potential adverse effects prior to starting a new medication improves patients’ knowledge of potential risks and does not lead to an increased incidence of those adverse effects (Lamb et al. 1994; Krska and Morecroft 2013). Therefore, adherence may improve if patients are specifically warned about, and given advice on how best to deal with, antidepressant-induced dizziness (that is, avoid standing up quickly).

Although the majority of adverse effects were not associated with discontinuation, there was some evidence of an association with the number of adverse effects reported. This suggests that the overall burden of adverse effects may be more important to tolerability than the majority of individual adverse effects. Additionally, the majority of individuals who discontinued antidepressant treatment cited adverse effects as the reason why. If this is the case, then reducing any adverse effect which is easily treatable may increase adherence. We acknowledge that other adverse effects not recorded at 2 weeks in this study may have an important role in discontinuation of antidepressant treatment—especially urinary problems and blurred vision, which have previously been associated with antidepressant discontinuation (Uher et al. 2009a).

There was a suggestion that four adverse effects (constipation, diarrhoea, nausea and drowsiness) may have a different impact on discontinuation between the treatment groups (*p* value for interaction between allocated treatment and adverse effect is <0.20). In the citalopram group, individuals reporting constipation or nausea had over twice the odds of discontinuing by 6 weeks. Conversely, individuals reporting drowsiness had a 60 % lower odds of discontinuing treatment from citalopram. The routine collection of discontinuation and adverse effect data that is now required for all RCTs will enable us to clarify the role these adverse effects may have in antidepressant discontinuation.

It appears that adverse effects induced by SSRIs or NARIs are only one factor important in treatment adherence. This is supported by a previous study of 265 depressed individuals receiving an SSRI which reported no evidence of an association between any single adverse effects, or overall adverse effect rating, with discontinuation (Warden et al. 2010).

#### Adverse effects over time

Previous studies and the BAP guidelines have reported that adverse effects tend to subside with time (Masand 2003; Anderson et al. 2008; Uher et al. 2009a). This may be due to the desensitisation of receptors that cause the adverse effects (Nutt and Glue 1991). Reports of half of the adverse effects, and the number of adverse effects reported, reduced between 2 and 12 weeks for both antidepressants. Additionally, all adverse effects, except diarrhoea, were reported less frequently at 12 weeks than at 6 weeks. Our results were not substantially different when we restricted our analysis to include only those who adhered to treatment. Therefore, the reduction in adverse effect reporting is not just due to fewer people taking the medication as the trial progressed.

Reports of diarrhoea tended to persist throughout the 12 weeks for individuals receiving reboxetine. Diarrhoea is regarded as a typical SSRI adverse effect and was reported much more frequently by individuals receiving citalopram in our study. Reports of diarrhoea from individuals receiving reboxetine may represent a physical symptom of depression rather than an adverse effect induced by reboxetine. The same may be true for individuals receiving citalopram and reporting sweating, a commonly reported adverse effect of NARIs, throughout the 12 weeks. Each physical symptom reported throughout the trial is likely due to a combination of being both a symptom of depression and an adverse effect.

Several of the adverse effects were reported less frequently at 2 weeks than at 6 weeks. Previous reports have suggested that the mode of administration does not introduce any bias, at least for mental health assessments (Evans et al. 2004). However, it is possible that the different methods of data collection at these two time points may explain this result. Alternatively, if these findings are replicated, it may encourage patients to know that after 6 weeks of treatment, reports of these adverse effects tend to reduce.

#### Implications and further research

The GENPOD study has allowed a detailed comparison between two pharmacologically different antidepressants, citalopram and reboxetine. Antidepressant-induced dizziness as well as the total number of adverse effects reported appeared to be important factors in discontinuation from antidepressant treatment. Greater information on the time course of specific physical symptoms will allow doctors to inform patients more clearly on the adverse effects they can expect throughout the duration of antidepressant treatment which may lead to greater adherence to treatment (Frank and Judge 2001; Lingam and Scott 2002). This has obvious and important clinical implications as individuals who discontinue antidepressant treatment prematurely may not benefit from treatment and are at a higher risk of relapse (Montgomery et al. 1993; Donoghue et al. 1996).

## Electronic supplementary material

Below is the link to the electronic supplementary material.

ESM 1 (PDF 630kb)
