# Supplementary material for: Adverse effects from antidepressant treatment: randomised controlled trial of 601 depressed individuals
Source: Psychopharmacology (Berl). 2014 Feb 13;231(15):2921–31. doi: 10.1007/s00213-014-3467-8 (PMC4099525; doi:10.1007/s00213-014-3467-8)
Supplement: Supplementary file 1 — (PDF 630kb) [file 213_2014_3467_MOESM1_ESM.pdf]

Article title: Adverse effects from antidepressant treatment: Findings from the GENPOD study

Journal: Psychopharmacology

Authors: Andrew A Crawford, Sarah Lewis, David Nutt, Tim J. Peters, Philip Cowen, Michael C. O'Donovan, Nicola Wiles, Glyn Lewis

Corresponding Author:

Andrew A Crawford

School of Social and Community Medicine, University of Bristol, Oakfield House

Oakfield Grove, Bristol, BS8 2BN, UK

Email: Andrew.crawford@bristol.ac.uk

Tel: +44 (0) 117 331 4007

Fax: +44 (0) 117 331 4026

Supplementary Table 1. Number of individuals reporting each adverse effect at baseline and at 2, 6 and 12 weeks after randomisation to citalopram

Adverse Effect	Baseline, n (%) N=298	2 week, n (%) N=284	6 week, n (%) N=273	12 week, n (%) N=253
A rapid heart beat	160 (54)	43 (15)	90 (33)	59 (23)
Tremor	75 (25)	47 (17)	55 (20)	41 (16)
Agitation	238 (80)	118 (42)	141 (52)	113 (45)
Dry mouth	170 (57)	157 (55)	148 (54)	97 (38)
Excessive sweating	140 (47)	106 (37)	124 (45)	99 (39)
Constipation	79 (27)	59 (21)	65 (24)	46 (18)
Diarrhoea	99 (33)	78 (27)	62 (23)	54 (21)
Felt sick or nauseous	191 (64)	111 (39)	105 (38)	84 (33)
Daytime drowsiness	257 (86)	166 (58)	193 (71)	170 (67)
Light headiness or dizziness	178 (60)	124 (44)	130 (48)	95 (38)
Hot flushes	114 (38)	102 (36)	112 (41)	83 (33)
Difficulty sleeping	273 (92)	170 (60)	186 (68)	158 (62)
Impotence	30 (38)	20 (26)	20 (27)	13 (19)
Difficulty Ejaculating	22 (31)	28 (40)	27 (36)	16 (24)
Mean number of adverse effects (sd)	6.6 (2.5)	4.5 (2.4)	5.2 (2.8)	4.3 (2.8)

For the male specific questions, the percentages have been calculated on the total number of males responding to each question. For impotence this was 79 males at baseline, 78 at 2 weeks, 75 at 6 weeks and 67 at 12 weeks; for difficulty ejaculating this was 72 males at baseline, 70 at 2 weeks, 75 at 6 weeks and 66 at 12 weeks.

Supplementary Table 2. Number of individuals reporting each adverse effect at baseline and at 2, 6 and 12 weeks after randomisation to reboxetine

Adverse Effect	Baseline, n (%) N=302	2 week, n (%) N=291	6 week, n (%) N=272	12 week, n (%) N=231
A rapid heart beat	167 (55)	83 (29)	126 (46)	79 (34)
Tremor	94 (31)	60 (21)	61 (22)	34 (15)
Agitation	244 (81)	156 (54)	178 (65)	120 (52)
Dry mouth	192 (63)	228 (78)	201 (74)	130 (56)
Excessive sweating	147 (49)	155 (53)	157 (58)	102 (44)
Constipation	89 (29)	135 (46)	140 (51.5)	81 (35)
Diarrhoea	76 (25)	30 (10)	26 (10)	30 (13)
Felt sick or nauseous	181 (60)	108 (37)	106 (39)	83 (36)
Daytime drowsiness	260 (86)	153 (53)	174 (64)	128 (55)
Light headiness or dizziness	190 (63)	168 (58)	144 (53)	92 (40)
Hot flushes	138 (46)	157 (54)	155 (57)	102 (44)
Difficulty sleeping	270 (89)	210 (72)	200 (73.5)	157 (68)
Impotence	19 (25)	39 (48)	28 (38)	19 (33)
Difficulty Ejaculating	14 (19)	36 (45)	29 (39)	13 (23)
Mean number of adverse effects (sd)	6.8 (2.4)	5.6 (2.3)	6.1 (2.6)	4.9 (2.8)

For the male specific questions, the percentages have been calculated on the total number of males responding to each question. For impotence this was 75 males at baseline, 82 at 2 weeks, 74 at 6 weeks and 57 at 12 weeks; for difficulty ejaculating this was 73 males at baseline, 80 at 2 weeks, 74 at 6 weeks and 57 at 12 weeks.

Supplementary Table 3. Time specific odds ratios or linear regression coefficients for the adverse effects where the effect of treatment varied with time

	2 Week			6 Week			12 Week		
**Adverse Effect**	**OR**	**95% CI**	***P***	**OR**	**95% CI**	***p***	**OR**	**95% CI**	***p***
Excessive Sweating	1.98	1.39, 2.74	<0.001	1.75	1.22, 2.50	0.002	1.19	0.82, 1.73	0.36
Diarrhoea	0.31	0.19, 0.50	<0.001	0.38	0.23, 0.63	<0.001	0.57	0.34, 0.95	0.03
Dizziness	1.81	1.29, 2.55	0.001	1.24	0.87, 1.77	0.24	1.00	0.68, 1.47	0.99
	**Coefficient**	**95% CI**	***P***	**Coefficient**	**95% CI**	***p***	**Coefficient**	**95% CI**	***p***
Number of adverse effects	1.08	0.72, 1.45	<0.001	0.90	0.49, 1.32	<0.001	0.41	-0.06, 0.88	0.08

Models are adjusted for baseline adverse effect, severity of depression and centre. An OR greater than 1 indicate that the adverse effect was more commonly reported by individuals receiving reboxetine than citalopram. A positive coefficient indicates a greater number of adverse effects reported by individuals receiving reboxetine.

Supplementary Table 4. Summary odds ratios reporting the effect of time on reporting adverse effects in a combined analysis, and stratified by allocated treatment group, from a repeated measures analysis at 0 and 2 weeks

Adverse Effect	Citalopram			Reboxetine			Combined		
	OR	95% CI	P	OR	95% CI	P	p value for interaction between time and treatment, *p*	OR	95% CI
Tremor	0.71	0.57, 0.90	0.005	0.65	0.51, 0.83	0.001	0.84	0.68	0.58, 0.81
Agitation	0.40	0.32, 0.51	<0.001	0.45	0.35, 0.57	<0.001	0.07	0.42	0.36, 0.50
Dry mouth	0.95	0.79, 1.14	0.60	1.50	1.23, 1.82	<0.001	<0.001	1.18	1.04, 1.35
Excessive sweating	0.77	0.63, 0.93	0.008	1.12	0.94, 1.33	0.22	0.005	0.94	0.83, 1.07
Constipation	0.79	0.63, 1.00	0.05	1.54	1.27, 1.87	<0.001	<0.001	1.18	1.03, 1.36
Diarrhoea	0.84	0.69, 1.03	0.09	0.51	0.38, 0.67	<0.001	0.004	0.69	0.59, 0.81
Nausea	0.56	0.46, 0.68	<0.001	0.58	0.48, 0.71	<0.001	0.65	0.57	0.50, 0.65
Dizziness	0.64	0.52, 0.78	<0.001	0.88	0.74, 1.06	0.19	0.03	0.76	0.66, 0.87
Daytime drowsiness	0.44	0.34, 0.56	<0.001	0.33	0.25, 0.45	<0.001	0.39	0.39	0.32, 0.47
Difficulty sleeping	0.25	0.17, 0.38	<0.001	0.50	0.39, 0.66	<0.001	0.003	0.38	0.30, 0.47
Hot Flushes	0.91	0.73, 1.12	0.37	1.23	1.02, 1.48	0.03	0.03	1.08	0.94, 1.24
Rapid heartbeat	0.30	0.22, 0.41	<0.001	0.50	0.40, 0.62	<0.001	0.002	0.40	0.34, 0.48
Impotence	0.64	0.40, 1.03	0.07	1.99	1.24, 3.17	0.004	<0.001	1.18	0.89, 1.57
Difficulty ejaculating	1.40	0.89, 2.20	0.15	3.00	1.48, 6.10	0.002	0.08	1.92	1.33, 2.78
	Coefficient	95% CI	P	Coefficient	95% CI	P	p value for interaction	coefficient	95%CI

							between time and treatment, *p*		
Number of Adverse Effects	-1.06	-1.22, -0.90	<0.001	-0.55	-0.72, -0.39	<0.001	<0.001	-0.80	-0.92, -0.67

The summary odds ratios indicate the effect of an increase in time of one week on reports of each adverse effect, during the first 2 weeks of treatment. Models are adjusted for severity of depression, centre and in the combined analysis, baseline adverse effect. An OR greater than 1 (or positive regression coefficient) indicates that reports of the adverse effect were more common in the first 2 weeks of receiving treatment than at baseline. The interaction reports the p value for the interaction between time (as a continuous variable) and allocated treatment group.

Supplementary Table 5. Summary odds ratios reporting the effect of time on reporting adverse effects in a combined analysis, and stratified by allocated treatment group from a repeated measures analysis at 2, 6 and 12 weeks for individuals adhering to treatment for at least 4 weeks

Adverse effect	Citalopram			Reboxetine			Combined		
	OR	95% CI	P	OR	95% CI	P	p value for interaction between time and treatment, *p*	OR	95%CI
Tremor	0.99	0.93, 1.05	0.74	0.95	0.88, 1.01	0.12	0.35	0.97	0.93, 1.02
Agitation	1.01	0.97, 1.05	0.71	1.00	0.96, 1.05	0.90	0.88	1.01	0.97, 1.04
Dry mouth	0.88	0.84, 0.93	<0.001	0.90	0.85, 0.95	<0.001	0.88	0.89	0.86, 0.92
Excessive sweating	1.01	0.96, 1.05	0.76	0.96	0.91, 1.01	0.13	0.17	0.97	0.95, 1.02
Constipation	0.98	0.92, 1.04	0.43	0.95	0.90, 1.01	0.11	0.61	0.97	0.93, 1.01
Diarrhoea	0.95	0.90, 1.00	0.05	1.02	0.93, 1.12	0.68	0.19	0.96	0.92, 1.01
Nausea	0.96	0.92, 1.00	0.07	0.96	0.91, 1.01	0.10	0.90	0.96	0.93, 0.99
Dizziness	0.95	0.91, 1.00	0.04	0.92	0.88, 0.97	0.002	0.27	0.94	0.91, 0.97
Daytime drowsiness	1.05	1.00, 1.10	0.06	1.00	0.95, 1.05	0.95	0.20	1.02	0.99, 1.06
Difficulty sleeping	1.01	0.96, 1.06	0.80	0.98	0.93, 1.04	0.54	0.45	1.00	0.96, 1.03
Hot Flushes	0.98	0.93, 1.03	0.38	0.96	0.91, 1.02	0.18	0.65	0.97	0.93, 1.01
Rapid heartbeat	1.04	0.98, 1.10	0.18	1.05	1.00, 1.11	0.06	0.69	1.05	1.01, 1.09
Impotence	0.93	0.82, 1.06	0.28	0.96	0.84, 1.09	0.52	0.75	0.95	0.87, 1.04
Difficulty ejaculating	0.82	0.70, 0.95	0.01	0.84	0.71, 1.00	0.05	0.69	0.83	0.74, 0.93
	Coefficient	95% CI	*P*	Coefficient	95% CI	*p*	p value for interaction between time and treatment, *p*	Coefficient	95%CI

Number of adverse effects	-0.03	-0.06, 0.00	0.07	-0.06	-0.10, -0.02	0.006	0.28	-0.04	-0.07, -0.02

The summary odds ratios indicate the effect of an increase in time of one week on reports of each adverse effect. Models are adjusted for severity of depression, centre and in the combined analysis, baseline adverse effect. An OR greater than 1 (or positive regression coefficient) indicates that the adverse effect increases with time. The interaction reports the p value for the interaction between time (as a continuous variable) and allocated treatment.

Supplementary Table 6. Odds ratios of discontinuing antidepressant treatment between 2 and 6 weeks by adverse effect at 2 weeks according to treatment allocation

Adverse Effect at 2 weeks	Citalopram				Reboxetine			
	N	OR	95% CI	p value	N	OR	95% CI	p value
Tremor	264	0.99	0.31, 3.12	0.99	248	1.60	0.74, 3.44	0.23
Agitation	264	0.94	0.40, 2.26	0.90	248	1.78	0.96, 3.29	0.07
Dry mouth	264	1.35	0.56, 3.26	0.51	247	0.78	0.36, 1.70	0.53
Excessive sweating	264	1.79	0.74, 4.31	0.20	248	1.28	0.70, 2.35	0.42
Constipation	264	2.82	1.06, 7.48	0.04	247	1.10	0.60, 2.00	0.76
Diarrhoea	264	0.75	0.27, 2.05	0.58	248	2.02	0.78, 5.19	0.15
Nausea	264	2.41	1.03, 5.67	0.04	248	1.08	0.58, 1.98	0.82
Dizziness	264	2.01	0.82, 4.93	0.13	248	1.78	0.93, 3.40	0.08
Daytime drowsiness	264	0.40	0.17, 0.94	0.04	248	0.94	0.52, 1.71	0.84
Difficulty sleeping	243	0.62	0.27, 1.44	0.27	248	1.07	0.53, 2.18	0.85
Hot flushes	264	1.28	0.51, 3.20	0.60	248	1.56	0.83, 2.92	0.17
Rapid heartbeat	264	0.68	0.19, 2.51	0.57	248	1.33	0.69, 2.55	0.40
Impotence	63	1.13	0.15, 8.71	0.90	58	0.81	0.24, 2.76	0.73
Difficulty ejaculating	36	1.78	0.29, 11.12	0.54	55	0.99	0.27, 3.59	0.99
Number of adverse effects	264	1.07	0.89, 1.29	0.48	246	1.14	0.99, 1.33	0.08

Odds ratios (OR) are estimates from logistic regression models. Models are adjusted for baseline physical symptom, severity of depression and centre. An OR greater than 1 indicates that the adverse effect is associated with a higher odds of discontinuation from antidepressant treatment between weeks 2 to 6 weeks. The interaction reports the p value for the interaction between adverse effect and allocated treatment.

Supplementary Figure 1. Modified version of the Toronto Side Effects Scale (TSES)

Symptoms

To begin, I am going to ask you some questions about symptoms you may have experienced since I saw you. When I read out each question, please tell me if you have or have not experienced the symptom during the last 2 weeks. If you have, I'd like to know on how many days you have experienced that symptom in the past 7 days.

During the last 2 weeks……

1. (a) Have you felt sick or nauseous?

No ☐1 **Yes** ☐2 ⟶ (b) On how many days during the last 7 days?

None ☐1 **1-3 days** ☐2 **4-7 days** ☐3

2. (a) Have you had a dry mouth?

No ☐1 **Yes** ☐2 ⟶ (b) On how many days during the last 7 days?

None ☐1 **1-3 days** ☐2 **4-7 days** ☐3

3.(a) Have you vomited?

No ☐1 **Yes** ☐2 ⟶ (b) On how many days during the last 7 days?

None ☐1 **1-3 days** ☐2 **4-7 days** ☐3

4.(a) Have you felt light headed or dizzy?

No ☐1 **Yes** ☐2 ⟶ (b) On how many days during the last 7 days?

None ☐1 **1-3 days** ☐2 **4-7 days** ☐3

5. (a) Have you had difficulty sleeping?

No ☐1 **Yes** ☐2 ⟶ (b) On how many days during the last 7 days?

None ☐1 **1-3 days** ☐2 **4-7 days** ☐3

6. (a) Have you been drowsy during the day?

No ☐1 **Yes** ☐2 ⟶ (b) On how many days during the last 7 days?

None ☐1 **1-3 days** ☐2 **4-7 days** ☐3

7. (a) Have you been agitated?

No ☐1 **Yes** ☐2 ——► (b) On how many days during the last 7 days?

None ☐1 **1-3 days** ☐2 **4-7 days** ☐3

8. (a) Have you sweated excessively?

No ☐1 **Yes** ☐2 ——► (b) On how many days during the last 7 days?

None ☐1 **1-3 days** ☐2 **4-7 days** ☐3

9. (a) Have you had tremor?

No ☐1 **Yes** ☐2 ——► (b) On how many days during the last 7 days?

None ☐1 **1-3 days** ☐2 **4-7 days** ☐3

During the last 2 weeks,……

10. (a) Have you had a rapid heart beat?

No ☐1 **Yes** ☐2 ——► (b) On how many days during the last 7 days?

None ☐1 **1-3 days** ☐2 **4-7 days** ☐3

11. (a) Have you had diarrhoea?

No ☐1 **Yes** ☐2 ——► (b) On how many days during the last 7 days?

None ☐1 **1-3 days** ☐2 **4-7 days** ☐3

12. (a) Have you been constipated?

No ☐1 **Yes** ☐2 ——► (b) On how many days during the last 7 days?

None ☐1 **1-3 days** ☐2 **4-7 days** ☐3

13. (a) Have you had hot flushes?

No ☐1 **Yes** ☐2 ——► (b) On how many days during the last 7 days?

None ☐1 **1-3 days** ☐2 **4-7 days** ☐3

Males only – sexual function

The next two questions ask about your sexual functioning during the last 2 weeks. These may not apply to you but again I will read each question to you. Please tell me if the question does not apply to you or whether you have or have not experienced these symptoms.

During the last 2 weeks,……

14. Have you had difficulty getting or maintaining an erection?

No ☐1 **Yes** ☐2 **Not applicable** ☐3

15. Have you had difficulty ejaculating?

No ☐1 **Yes** ☐2 **Not applicable** ☐3

Males and Females - Other physical symptoms

16. (a) Have you had any other physical symptoms since you started taking your tablets 2 weeks ago that I have not mentioned already?

No ☐1 **Yes** ☐2 ──► (b) Could you please describe these briefly?

(c) On how many days during the last 7 days?

None ☐1 **1-3 days** ☐2 **4-7 days** ☐3
